# The complete chloroplast genome sequence of *Garcinia oblongifolia* (Clusiaceae)

**DOI:** 10.1080/23802359.2020.1810162

**Published:** 2020-08-25

**Authors:** Xiang Ma, Weifang Chen, Liang Tang

**Affiliations:** a Key Laboratory of Tropical Biological Resources of Ministry of Education, School of Life and Pharmaceutical Sciences, Hainan University, Haikou, China; b College of Ecology and Environment, Hainan University, Haikou, China; c Center for Terrestrial Biodiversity of the South China Sea, Hainan University, Haikou, China

**Keywords:** *Garcinia oblongifolia*, complete chloroplast genome, Clusiaceae

## Abstract

*Garcinia oblongifolia* Champ. ex Benth is rich in bioactive molecules with immense remedial qualities and has pharmaceutical potentials. In this study, we reported the complete chloroplast genome (cpDNA) of *G. oblongifolia*, which is 156,577 bp in length, including a large single copy region (LSC) of 85,393 bp, a small single copy region (SSC) of 17,064 bp, and a pair of inverted repeats (IR) regions of 27,060 bp inserted between LSC and SSC. 129 genes are encoded, including 83 protein-encoding genes, 8 ribosomal RNA genes, and 36 transfer RNA genes. The overall GC content of the chloroplast genome is 36.2%, wherein the corresponding values in the LSC, SSC and IR regions are 33.6%, 30.3%, and 42.2%, respectively. Phylogenetic analysis showed that *G. oblongifolia* is sister to *G. gummi-gutta* with strong bootstrap support.

*Garcinia oblongifolia* Champ. ex Benth (Clusiaceae) is a small tree rich in variety of bioactive molecules, including bioflavonoids, xanthones, and benzophenones, which have potent anticancer, antioxidant, anti-inflammatory and anti-microbial activities (Li et al. 2016). It is distributed in tropical regions of China, mainly in Guangxi, Guangdong and Hainan provinces. Its fruit is edible and the seed, bark and timber are useful industrial materials (Liu et al. 2016). Despite extensive studies on the pharmaceutical and nutritional components derived from *G*. *oblongifolia*, the genetic information for this species remains quite limited.

In this study, the complete plastome sequence of *G. oblongifolia* was reported and characterized. The leaves of *G. oblongifolia* were collected from one individual in Jinniu Park, Haikou, China (N 20°00′45″, E 110°18′55″), the voucher specimen of which was deposited at the Herbarium of Tropical Plant Research of Hainan University, with the number of GOJN1920. Total genomic DNA was extracted from silica gel-dried leaves following the modified CTAB method (Doyle and Doyle 1987). A genomic DNA library with an insert size of 400 bp was constructed and then sequenced by an Illumina HiSeq 2500 system at JINTAI Biotech (Guangzhou, China). Approximately, 9.2 GB paired-end sequencing data were generated after removing adapters and low-quality reads by fastp software (Chen et al. 2018), with a coverage of 403. Chloroplast genome of *G*. *oblongifolia* was assembled with GetOrganelle v1.6.2 (Jin et al. 2019) and annotated using the online tool DOGMA (Wyman et al. 2004). The complete chloroplast genome was submitted to GenBank (accession number: MT726019).

The chloroplast genome of *G*. *oblongifolia* shows a typical quadripartite structure of 156,577 bp in full length, consisting of a large single copy region (LSC) of 85,393 bp, a small single copy region (SSC) of 17,064 bp, and a pair of inverted repeats (IR) regions of 27,060 bp inserted between LSC and SSC. The total GC content of plastome is 36.2%, with the corresponding values of 33.6%, 30.3%, and 42.2% in the LSC, SSC, and IR regions respectively. The chloroplast genome comprises 129 genes, including 83 protein-coding genes, 8 ribosomal RNA genes, and 36 transfer RNA genes. 18 genes occurred in the IR region have two copies, including 7 protein-coding genes, 7 tRNA genes, and 4 rRNA genes. There are 111 unique genes, among which 17 genes have one intron, and two genes, *rps12* and *clp*P, have two introns.

To analyze the phylogenetic position of *G*. *oblongifolia*, complete chloroplast genomes of 31 species belonging to the order Malpighiales were retrieved from Genbank. *Averrhoa carambola* and *Cephalotus follicularis* were used as outgroups. The chloroplast genome alignment was constructed using MAFFT online service (Katoh and Standley 2013). Phylogenetic analysis was performed based on maximum likelihood (ML) method implemented in IQ-TREE 1.6.12 (Nguyen et al. 2015), with TVM + F+R3 selected as the best-fit nucleotide substitution model by ModelFinder (Kalyaanamoorthy et al. 2017). Four *Garcinia* species form a highly supported monophyletic group, in which *G*. *oblongifolia* is sister to *G. gummi-gutta* with bootstrap support of 100 (Figure 1). The completion and characterization of the complete plastid genome sequence in this study provided helpful molecular resource for future phylogenetic and evolutionary studies of the valuable tree *G*. *oblongifolia*.

**Figure 1.**
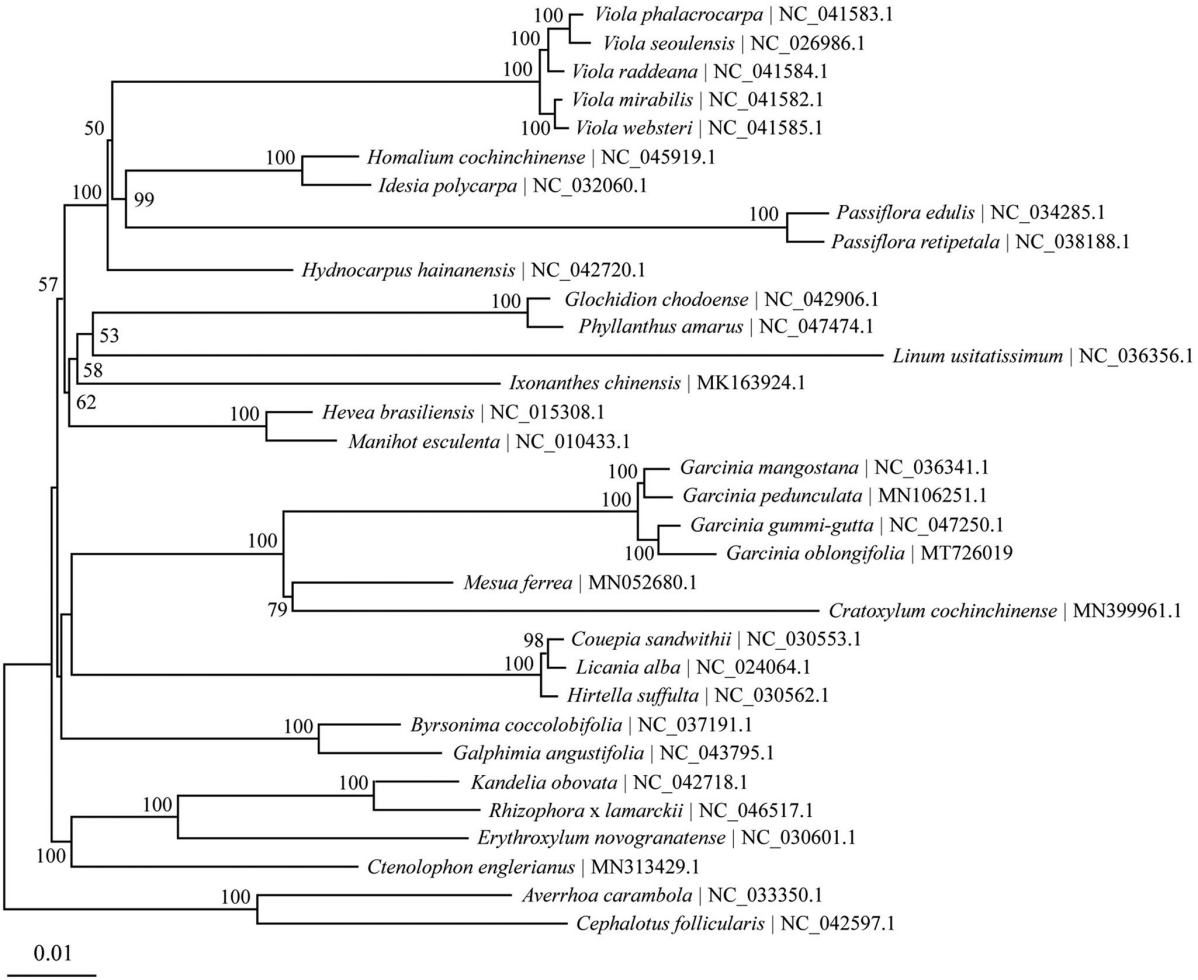
The Maximum-Likelihood (ML) tree based on 31 chloroplast genomes in order Malpighiales together with *Averrhoa carambola* and *Cephalotus follicularis* as the outgroups. Numbers above branches or near interior nodes are bootstrap support values based on 1000 replicates. Bootstrap support values lower than 50% are not shown.

## Data Availability

The data that support the findings of this study are openly available in NCBI at https://www.ncbi.nlm.nih.gov/, reference number [MT726019], or available from the corresponding author.
